# Beyond pain: modeling decision-making deficits in chronic pain

**DOI:** 10.3389/fnbeh.2014.00263

**Published:** 2014-08-01

**Authors:** Leonardo Emanuel Hess, Ariel Haimovici, Miguel Angel Muñoz, Pedro Montoya

**Affiliations:** ^1^Training and Research for Argentina Medical Association (CIMA), Faculty of Medical Sciences, National University of Rosario (UNR)Rosario, Santa Fe, Argentina; ^2^Research Institute on Health Sciences (IUNICS), University of Balearic Islands (UIB)Palma, Spain; ^3^Departamento de Física, Facultad de Ciencias Exactas y Naturales, Universidad de Buenos AiresFísica, Buenos Aires, Argentina; ^4^Consejo Nacional de Investigaciones Científicas y Tecnológicas (CONICET)Buenos Aires, Argentina; ^5^Departamento de Personalidad, Evaluación y Tratamientos Psicológicos, University of GranadaGranada, Spain

**Keywords:** chronic pain, decision-making, modeling, cognition, emotion

## Abstract

Risky decision-making seems to be markedly disrupted in patients with chronic pain, probably due to the high cost that impose pain and negative mood on executive control functions. Patients’ behavioral performance on decision-making tasks such as the Iowa Gambling Task (IGT) is characterized by selecting cards more frequently from disadvantageous than from advantageous decks, and by switching often between competing responses in comparison with healthy controls (HCs). In the present study, we developed a simple heuristic model to simulate individuals’ choice behavior by varying the level of decision randomness and the importance given to gains and losses. The findings revealed that the model was able to differentiate the behavioral performance of patients with chronic pain and HCs at the group, as well as at the individual level. The best fit of the model in patients with chronic pain was yielded when decisions were not based on previous choices and when gains were considered more relevant than losses. By contrast, the best account of the available data in HCs was obtained when decisions were based on previous experiences and losses loomed larger than gains. In conclusion, our model seems to provide useful information to measure each individual participant extensively, and to deal with the data on a participant-by-participant basis.

## Introduction

Pain is a dynamical (Foss et al., 2006) and highly attention-demanding sensory phenomenon (Eccleston and Crombez, 1999) which requires cognitive processing such as learning, recall of past experiences and active decision making (Keefe et al., 2001; Apkarian et al., 2004; Bechara and Damasio, 2005; Montoya et al., 2005; Solberg Nes et al., 2009; Abeare et al., 2010). Moreover, it is known that cognitive interventions including distraction, hypnosis, or mindfulness may have analgesic effects and that cognitive functioning seems to be markedly disrupted in patients with chronic pain (Must et al., 2006; Moriarty et al., 2011). In this sense, neural systems involved in cognition and pain seem to share an inherent overlap and may modulate one another reciprocally. Traditionally, emotion regulation and executive control in chronic pain has been studied by using statistic behavioral measures such as self-report questionnaires (Beck et al., 1961; Spielberger et al., 1970) and neuropsychological test batteries (Golden, 1978; Petrides et al., 1993; Bechara et al., 1994, 1997). This approach, however, is not adequate to study dynamical properties of the interaction between emotion and cognition in pain and, to our knowledge, moment-to-moment changes in such behaviors have remained unexplored in chronic pain states.

One of the most influential tools for the study of the dynamic of emotional processes involved in real-life decision-making is the Iowa Gambling Task (IGT) developed by Bechara and colleagues (Petrides et al., 1993; Bechara et al., 1996, 1997; Brand et al., 2007). This task requires that participants draw cards from one of four decks with the goal of winning as much money as possible. Each card is previously associated with the gain of specific amounts of money, and choices of some cards are also followed by loss of money. Overall, choosing from two of the decks (usually called “disadvantageous” decks) causes subjects to gain and to lose large amounts of money, whereas choosing from the other two (usually called “advantageous” decks) results in smaller gains and losses of money. Thus, the IGT has been developed to capture the elements of risk, persistence in wrong decisions and punishment which guide decision-making in real-life situations. According with Bechara and Damasio (2005), somatic markers such as autonomic responses to anticipation of choices during IGT performance can influence decision-making under uncertainty in healthy individuals, suggesting that emotion-guided reasoning facilitates decision-making processes.

Although the inter-subject variability on IGT performance is high, findings are very robust and mathematical modeling has already provided relevant information about the analysis and prediction of individual’s choice behavior on such risky decision-making tasks. In this sense, reinforcement learning models such as expectancy valence and prospect valence learning have been recently used to quantitatively characterize human performance on IGT (Worthy et al., 2013). The basic assumptions underpinning these models is that expected reward values for each option are determined by outcomes from past decisions, and that the probability of selecting options with high expected rewards is higher than the probability of selecting options with low expected rewards. Several variations of these models have been already compared in terms of level of predictability of card selection sequences (Steingroever et al., 2013; Worthy et al., 2013), and limitations of fitting at the individual level have been discussed (Wetzels et al., 2010). Nevertheless, there seems to be some fundamental features of the general strategies during decision-making in IGT. Thus, for instance, decisions on the IGT do not appear to be based on “expected long-term results” or on final net balance, but rather on frequency of gains and losses (Horstmann et al., 2012). Moreover, selections in the IGT seem to be predominantly characterized by the interaction of underlying psychological processes involved in the weighting of gains vs. losses, memory for past payoffs, and response consistency (Wetzels et al., 2010). In addition, a recent animal study has shown that a combination of individual traits, namely risk taking, reward seeking, behavioral inflexibility, and motor impulsivity, could be also highly predictive of performance on IGT (Rivalan et al., 2013).

Previous findings indicated that chronic pain patients are unable to develop an advantageous strategy and that they are less persistent in their choices, switching more often between competing responses than healthy controls (HCs) in the IGT (Apkarian et al., 2004; Verdejo-García et al., 2009; Walteros et al., 2011). Accordingly, the aim of the present work was to assess if a simplified version of previous models would be able to discriminate between patients and healthy individuals on IGT performance. For this purpose, the main assumption of our model was that risky decision-making performance on the IGT would be modulated by both cognitive (decision is guided by past experiences) and affective components (decision is guided by the relevance of gains and losses).

## Materials and methods

### Subjects

Empirical data of the present study were previously published by our group (Berry and Fristedt, 1985). The sample was composed by 15 patients with chronic pain fibromyalgia (FM) for at least 6 months (mean age 50.4 years, SD = 4.6) and 15 healthy volunteers (mean age 49.0 years, SD = 6.7). All participants underwent an extensive medical and psychological interview, including assessment of mood state (depression, anxiety) through self-report questionnaires and a standardized neuropsychological test battery to assess cognitive flexibility and resistance to interference (Stroop Interference test and six WAIS subscales, described in Walteros et al. (2011). Participants had no history of head trauma, substance abuse, pathological gambling or any other psychopathological disorder.

Patients were verbally informed about the details of the study. A specifically designed patient information leaflet was also given, and after agreeing to participate, each patient provided written consent. The study was in accordance with the Declaration of Helsinki and was approved by the local ethics board (reference: #IB833/07PI).

### The Iowa Gambling Task (IGT)

The task consisted of 100 trials in which participants were asked to select one card from one of four decks. The task was programmed to award different amounts of money (rewards) after each card selection and to deliver monetary losses of different amounts (punishments) in specific trials. Two decks were programmed to deliver high amounts of monetary gains and losses (disadvantageous decks), whereas the other two decks were programmed to deliver low amounts of monetary gains and losses (advantageous decks).

Participants were told that the goal of the task was to gain and to avoid losing as much money as possible, and that they were free to switch from any deck to another as often as they wished. No information was given about the existence of advantageous decks or about how much time they should play the game, although the task stopped automatically after 100 trials. Participants received a loan of 2000 € (play money) at the beginning of the task. Behavioral performance was analyzed by calculating the number of choices for the two types of decks (advantageous vs. disadvantageous) within each block of 20 trials. Net scores were obtained by subtracting the number of disadvantageous from the number of advantageous choices for each block according to the standardized method described previously (Bechara et al., 1997). The sequence order of the cards for each deck was defined previously and was the same for all participants. A persistence index was obtained by computing the total number of trials in which the participant was consecutively choosing cards from the same deck.

### Mathematical modeling

From a mathematical perspective, the IGT is a so-called four-armed bandit problem (Berry and Fristedt, 1985; Steyvers et al., 2009), a case of reinforcement learning problem in which participants learn the more appropriate behavior by choosing actions and readjusting performance through feedback obtained from their own actions. In order to find the most appropriate strategy in the IGT, it has been suggested that exploratory behavior should be guided by motivational characteristics of the task such as the distribution of reward rates (Steyvers et al., 2009) and the weight of gains and losses (Wetzels et al., 2010). In the present study, we have analyzed if a simple heuristic model would be able to capture the process of readjusting the behavior (*V*), defined as the ability to choose a specific deck and to modify this decision after choosing a card. In our model, behavior update after each trial was given by the following equation:

(1) $$V_{k}=\left(1-\rho \right)W+\rho L$$

where *W* was the amount gained and *L* the amount lost by choosing the deck *k*. The free parameter *ρ* varied according to importance given to gains and losses (weight of gains and losses). Thus, decision-making was biased by gains for small *ρ* values and by losses for large *ρ* values. At each time, probability of choosing a deck *k* was given by:

(2) $$P_{k}\left(t+1\right)=\frac{\text{exp}\left(V_{k}(t)/T\right)}{\sum_{k}\text{exp}\left(V_{k}(t)/T\right)}$$

where *T* was the degree of randomness used by the participants to make decisions. Thus, *T* was a random number from a uniform distribution between 0 (indicating decision guided by the last experience with the *k* deck) and *T*_max_ (indicating decision guided by a high level of randomness).

According with model predictions, persistent behavior (choosing cards from the same deck) should be reached when losses are considered more important than gains (high values of *ρ*) and decision randomness decreases (low values of *T*_max_). By contrast, less persistent behavior would appear when gains are subjectively considered more important than losses (low values of *ρ*) and decision randomness increases (high values of *T*_max_). The model was computed for different parameters of *ρ* and *T*_max_ by using the same sequences of rewards and losses as reported in our previous work (Walteros et al., 2011). Moreover, results from our mathematical model were fitted to those experimental data. Twenty time series were obtained for different *ρ* values between 0 and 1. The autocorrelation function of these time series was also used to capture the persistence behavior in our mathematical model. At the individual level, calculations of persistence level were used to find out those values of *ρ* and *T*_max_ parameters that best fit to subject’s performance.

## Results

**Figure 1:**
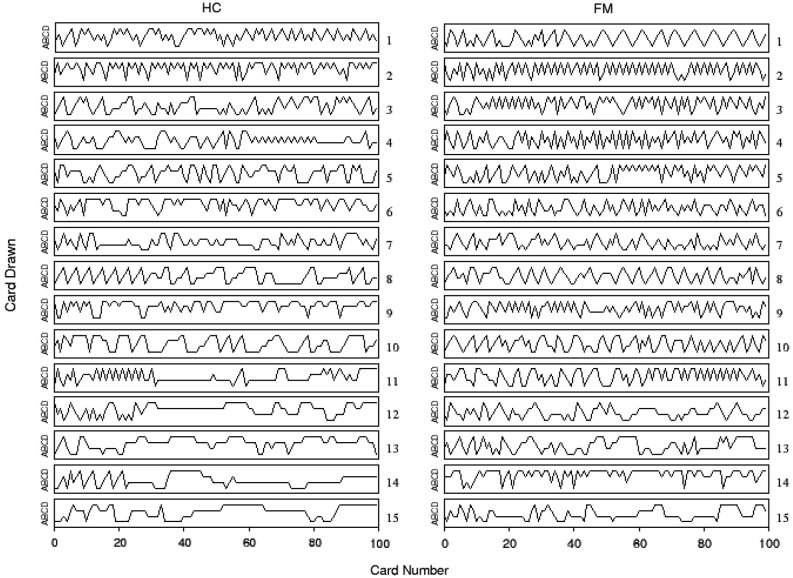
**Individual performance on the IGT for chronic pain patients fibromyalgia (FM) and healthy controls (HCs).** Time series of card choices were ordered according with their persistence in selecting cards from the same deck: the first row (subject 1) corresponds to the less persistent subject and the last one (subject 15) to the most persistent subject. Labels A and B correspond to advantageous decks, while C and D refer to disadvantageous ones.

Figure 1 illustrates the observed sequence of deck choices for each HC and chronic pain patient FM during the original experiment (Walteros et al., 2011). A visual examination of these data reveals that choices appeared to be less persistent in chronic pain patients than in healthy subjects. In particular, a significant group difference was yielded on the persistence index (*F*_(1,28)_ = 8.09, *p* < 0.01), showing that HCs displayed more persistent behavior (mean = 1.90, SD = 0.75) than chronic pain patients (mean = 1.27, SD = 0.37). Moreover, although patients with chronic pain showed more depression (*t*_(28)_ = 6.1, *p* < 0.001) and anxiety (*t*_(28)_ = 5.8, *p* < 0.001) than HCs, group difference on the persistence index was still significant after controlling for these mood variables (*F*_(1,28)_ = 7.75,* p* < 0.01). No significant group differences were observed on cognitive flexibility and resistance to interference as measured by the standardized neuropsychological test battery (see Table 1 in Walteros et al., 2011).

**Figure 2:**
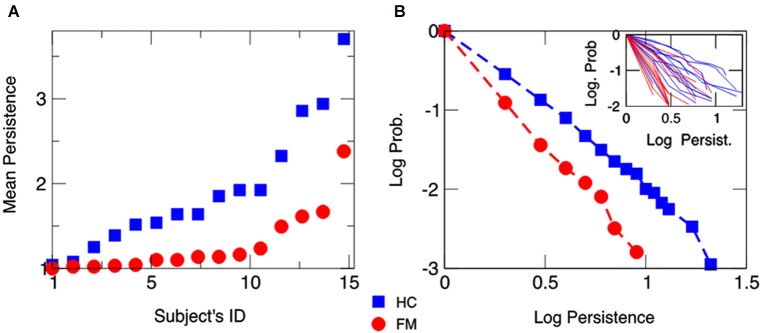
**Mean behavioral persistence on the IGT for chronic pain patients fibromyalgia (FM) and healthy controls (HCs). (Panel A)** Mean number of trials in which a participant was consecutively drawing cards from the same deck throughout the task. Subjects’ IDs are identical as in Figure 1. **(Panel B)** Average CDF of the persistence behavior observed in chronic pain patients (FM) and HCs. The average CDF in chronic pain patients has a steeper slope than in HCs, showing that patients are less persistent in drawing cards than HCs. The inset displays individuals’ CDFs.

Figure 2A also displays the mean persistence index calculated for each participant, and Figure 2B shows the average cumulative distribution function (CDF) of persistent behavior calculated separated by each group and each participant. Data indicated that the slope of the CDF was steeper in chronic pain patients than in HCs (see also inset of Figure 2A for individual CDFs), corroborating that choice behavior was less persistent in chronic pain patients than in HCs.

**Figure 3:**
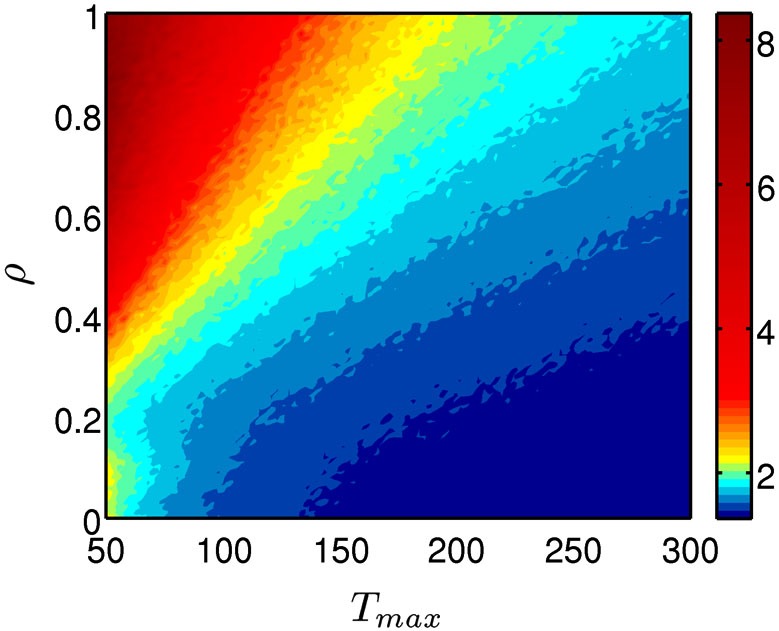
**Persistence of the choice behavior predicted by the mathematical model**. The model was computed for different values of for different values of *ρ* (importance given to gains or losses) and *T*_max_ parameters (degree of decision randomness) and the resulted persistence level was plotted by using a color code, with red indicating high probability and blue high probability of keep selecting from the same deck.

**Figure 4:**
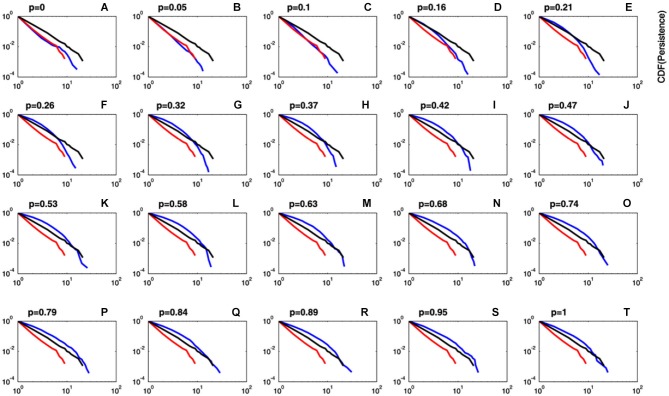
**Comparison between predicted and observed cumulative distribution functions of persistence.** Plots of the CDF of the predicted persistence (blue lines) at different values of *ρ* (importance given to gains or losses) and a fixed value of *T*_max_ = 50 (degree of decision randomness). For comparison purposes, CDFs for observed data in chronic pain patients (red lines) and HCs (black lines) are also displayed. It can be observed that CDF for predicted persistence by the model at *ρ* values close to zero (blue lines in plots **A–C**) perfectly fits data in chronic pain patients (red line). Plots of the CDF computed by the model were close to data in HCs (black lines) for *ρ* values greater than 0.15 (plots **D–T**).

**Figure 5:**
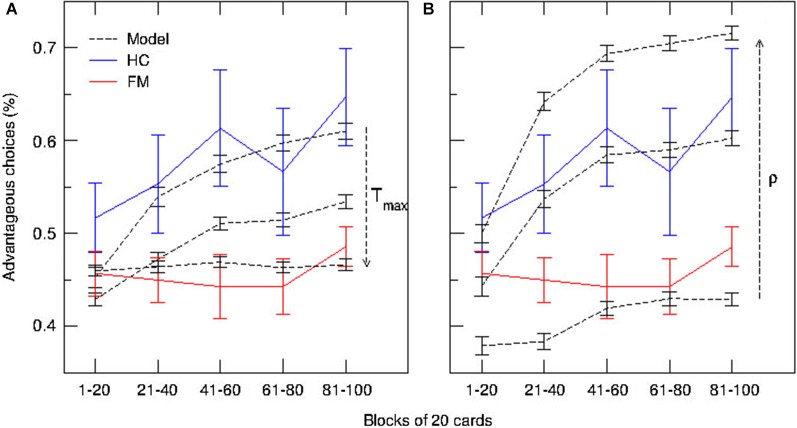
**Predictions of the model and behavioral performance of patients with chronic pain and healthy controls on the IGT over time.** Blue and red lines represent the median used to compute proportions of advantageous and disadvantageous choices for each block of 20 trials in patients with chronic pain (FM) and HCs, respectively. **(A)** Simulated time-course of choices at three different values of *T*_max_ (degree of decision randomness) (dashed lines). **(B)** Simulated time-course of choices at three different values of *ρ* (importance given to gains or losses) (dashed lines).

The persistence index of the choice behavior computed by our mathematical model for different values of *ρ* and *T*_max_ parameters is displayed in Figure 3. As it was predicted, high values of *ρ* and low values of *T*_max_ led to more persistent behavior. Figure 4 displays the average CDFs from data predicted by our model for several *ρ* values and a given *T* value (*T*_max_ = 50) in comparison with CDFs from observed behavior choices in chronic pain patients and HCs. Figure 5 displays the behavioral performance of patients with chronic pain and HCs for each block of 20 trials on the IGT, as well as the predicted performance of our model by three values of *T*_max_ (Figure 5A) and *ρ* parameters (Figure 5B).

**Figure 6:**
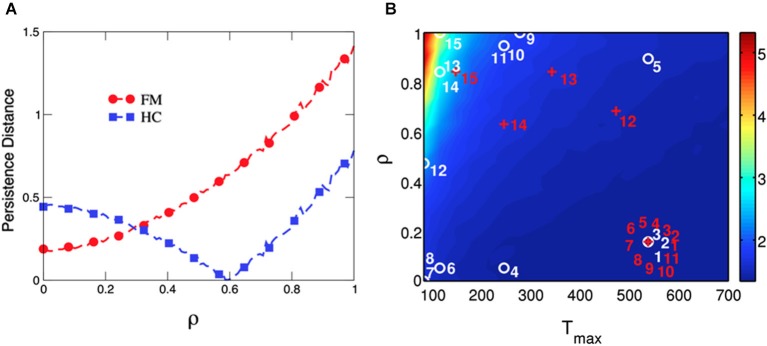
**Goodness-of-fit of the model computed as the distance between predicted and observed behavioral persistence for chronic pain patients and healthy controls at the group- and the individual-level.** Data modeling of chronic pain patients (FM) show that the distance between predicted and observed data is minimized at *ρ* = 0, whereas the distance is minimal at *ρ* = 0.6 for HCs **(Panel A)**. The parameter *T*_max_ (degree of decision randomness) was held constant at 50, as in Figure 4. The model is able to separate both groups by fitting the persistence of each single subject in the space of the free parameters in our model (*ρ* and *T*_max_) **(Panel B)**. The labels for chronic pain patients (numbered red crosses) and HCs (numbered white circles) are placed where the difference between predicted and observed persistence is minimized. The persistent behavior of most chronic pain patients (numbered from 1 to 11 in red) is best reproduced by the model when *T*_max_ is high (ramdom decision) and *ρ* is low (more importance is given to gains than to losses). On the other hand, performance of most HCs is best reproduced with lower *T*_max_ (decision guided by the last experience) and higher values of *ρ* (more importance is given to losses than to gains).

Finally, the average distance between persistence values predicted by the model and those collected from behavioral performance in both groups were computed to test the goodness-of-fit of our model at the group-level (Figure 6A). Results indicated that distance between predicted and observed data was minimized at *ρ* = 0.6 in HCs, whereas distance was minimal at *ρ* close to 0 in chronic pain patients. A similar result was obtained when the best fitted distances between predicted and observed data were computed for each participant to visualize the goodness-of-fit of our model at the individual-level (Figure 6B). Our mathematical model provides the best account for the observed data in HCs when *T*_max_ levels were low (decision guided by previous experience) and *ρ* values were high (losses loom larger than gains), corresponding to high persistent choice behavior in the IGT (Figure 6B, numbers from 4 to 15 in white). By contrast, high *T*_max_ levels (high decision randomness) and low *ρ* values (gains are more relevant than losses) were the parameters of our mathematical model that best fitted behavioral performance in most patients with chronic pain (Figure 6, numbers from 1 to 11 in red).

## Discussion

The objective of this study was to develop a mathematical heuristic model to simulate individual’s behavioral performance on a risky decision-making test such as the IGT. Furthermore, the study was aimed to test the feasibility of this model to fit performance on the IGT in chronic pain patients at the group, as well as at the individual level.

The IGT has been extensively used along with other neuropsychological tests to assess cognitive functions involved in real-life decision making in patients with ventromedial and orbitofrontal cortex damage (Bechara et al., 1994, 1996), or psychiatric diseases (i.e., obsessive compulsive disorder, schizophrenia, substance abuse, pathological gambling) (Buelow and Suhr, 2009). Basically, results indicated that these patients display a significant impaired risky behavior, characterized by choices that yield high immediate gains in spite of higher future losses (disadvantageous decks) (Buelow and Suhr, 2009). These patients continue to perseverate with choices from disadvantageous decks throughout the task, sometimes even though they know that they are losing money overall. Similarly, patients with chronic pain (Apkarian et al., 2004; Verdejo-García et al., 2009; Walteros et al., 2011) and affective disorders such as depression (Must et al., 2006) are also unable to develop an advantageous strategy in the IGT, suggesting that pain and negative mood might impose a high cost on executive control, undermining mainly affective processes involved in decision-making. Nevertheless, contrary to patients with psychopathological or neurological disorders, patients with chronic pain seem to be significantly less persistent in their choices, switching often between competing responses, and thus exhibiting a more random choice behavior than HCs and patients with psychiatric or neurological disorders.

Indeed, behavioral performance on such risky decision-making tasks is supposed to be influenced by “cold” cognitive and “hot” affective processing (Buelow and Suhr, 2009). Cognitive processing may involve the knowledge of risk/benefit ratios, the ability to retrieve them from memory and to hold them in mind while comparing and contrasting results with previous experience (working memory), whereas affective processing would involve emotional responses to selected options (Seguin et al., 2007). Based on these assumptions, we developed a simple mathematical model in which choice b ehavior on the IGT was modulated by both a cognitive (persistence of past experience, *T* parameter) and an affective component of risky decision-making (relevance of gains and losses, *ρ* parameter). According with model predictions, behavior became more persistent when decisions were based on previous experience (low values of *T*_max_) and losses were considered more important than gains (high values of *ρ*). By contrast, less persistent behavior appeared when decision randomness increased (high values of *T*_max_) and gains were considered more important than losses (low values of *ρ*). The goodness-of-fit of our model to data in chronic pain patients and HCs was evaluated by using the distance between predicted and observed values of persistence during the IGT, thus providing a numerical measure of the accuracy of the prediction at the group and at the individual level. Our results indicated that the best fit of the model in patients with chronic pain was yielded when decisions were not based on previous experiences (low behavioral persistence) and they were guided by the relevance of gains over losses. By contrast, our model provided the best account of the available data in HCs when decisions were based on previous experiences and losses loomed larger than gains. Thus, our model was sufficient to provide the best fit of behavioral performance deficits on the IGT in chronic pain patients.

In this sense, our findings are in agreement with previous mathematical models of IGT performance using Bayesian hierarchical estimation procedures (Wetzels et al., 2010; Rivalan et al., 2013; Worthy et al., 2013). All these previous models were also based on the assumption than individuals’ choice behavior on the IGT could be influenced by a combination of cognitive and affective factors. In this sense, it has been shown that choices in risky decision-making tasks are predominantly characterized by the interaction of psychological processes (weighting of gains vs. losses, memory for past payoffs, and response consistency) (Wetzels et al., 2010), or a combination of individual behavioral traits (risk taking, reward seeking, behavioral inflexibility, and motor impulsivity) (Rivalan et al., 2013). Furthermore, some of these computational models were based on the assumption that risk and reward seeking, together with behavioral inflexibility are hallmarks of poor decision-making occurring in some mental disorders such as attention deficit hyperactivity disorders, personality disorders, substance abuse, pathological gambling or mania (Rivalan et al., 2013). By contrast, we were based on previous experimental data indicating that chronic pain patients were unable to develop an advantageous strategy in the IGT, because they were less persistent in their choices, switching more often between competing responses than HCs. These findings were in agreement with previous results indicating that chronic pain may be associated with specific cognitive impairments, probably related to the processing of sensory, cognitive and affective information arising from the body (Eccleston and Crombez, 1999; Keefe et al., 2001; Apkarian et al., 2004; Montoya et al., 2005; Walteros et al., 2011). Thus, we reason that chronic pain probably imposes a high cost on decision-making, undermining mainly affective-based decision-making and reducing the availability of the limited attentional resources for parallel processing of other information than pain (Eccleston and Crombez, 1999; Montoya et al., 2005; Walteros et al., 2011). In this sense, it could be that poor behavioral performance in chronic pain could result from different combinations of predisposing traits and neurocognitive endophenotypes than in psychiatric and neurological disorders. Future research should explore the role of different neuropsychological determinants of poor decision-making as a potential risk factor for developing chronic pain by integrating and comparing multiple behavioral and neurophysiological (EEG, fMRI) measures in computational modeling.

In summary, our model was also able to differentiate between healthy participants and chronic pain patients at the group- and the individual-level. The model developed here is extremely simple to implement and fast to compute, and may be a useful surrogate for the optimal recursive decision process in some niche applications. Moreover, it could be used to explore a range of different tasks in order to select one that allows researchers to extract relatively large amount of information from a single participant’s choice performance. In conclusion, our model seems to provide useful information to measure each individual participant extensively, and to deal with the data on a participant-by-participant basis.

## Conflict of interest statement

The authors declare that the research was conducted in the absence of any commercial or financial relationships that could be construed as a potential conflict of interest.
